# Comment on: “Attempting to Separate Placebo Effects from Exercise in Chronic Pain: A Systematic Review and Meta-analysis”

**DOI:** 10.1007/s40279-021-01621-8

**Published:** 2021-12-21

**Authors:** Maximilian Koeppel, Stefan Peters, Gerhard Huber, Friederike Rosenberger, Joachim Wiskemann

**Affiliations:** 1 Working Group Exercise Oncology, Division of Medical Oncology, National Center for Tumor Diseases Heidelberg (NCT Heidelberg), 69120 Heidelberg, Germany; 2 German Association for Health-Related Fitness and Exercise Therapy (DVGS e.V.), Hürth-Efferen, Germany; 3 Institute of Sports and Sport Science, Heidelberg University, Heidelberg, Germany

Dear Editor,

With great interest, we read the recently published systematic review and meta-analysis by Miller et al. [1]. We appreciate the endeavor presented by the authors to investigate the efficacy of exercise regarding chronic pain, as it is an epidemiologically highly relevant topic. Furthermore, we believe that it is important for exercise therapy research to engage in placebo-controlled trials. Thus, we completely agree with the authors that a lack of scientific rigor can often be observed in exercise science; however, we strongly disagree with the key point of the paper, namely that exercise training for chronic pain is just as effective as non-exercise placebo treatments.

In this article, the authors cannot find statistically significant superiority in favor of exercise interventions in (non-exercise) placebo-controlled trials; however, the “absence of evidence”, meaning being unable to reach statistical significance to reject the null hypothesis does not equal “evidence for the absence” of an effect [2, 3]. In the meta-analysis of Miller et al. [1], the reason for a non-significant result appears to be the lack of statistical power. This issue becomes more obvious when we look into its effect size estimation. The authors calculated a mean effect of 0.94, which would be considered a substantial effect in favor of exercise in most contexts and effect size conventions (e.g., [4]). Despite the large effect, the analysis failed to reach statistical significance, which can be attributed to the small number of included trials (*n* = 4), their considerable effect heterogeneity (*I*^2^ = 92.4%), and the small sample sizes, considering that three out of four studies have a total of 40 participants (i.e., maximum of 20 per group). In addition, the large 95% confidence interval (CI), from − 0.17 to + 2.06, supports low statistical power. At this point, we need to ask to what extent this interval makes sense. If we apply the common language effect size index by McGraw and Wong [5] to the boundaries of the 95% CI, the lower bound would mean that when comparing a randomly drawn person from both the exercise and the placebo control group, the results would show the placebo control group as superior in approximately 55 of 100 cases. If we look at the upper bound of the 95% CI, the random person drawn from the exercise group would outperform the person from the placebo group in almost every comparison (approximately 93%). Consequently, there is hardly any meaningful information to be derived from the 95% CI. In order to improve the interpretation of these results, Dent and Raftery [6] can be helpful because they take the direction of the effect and the uncertainty of the effect estimate into consideration when interpreting their results. According to that approach, the results from Miller et al. [1] need to be interpreted as “inconclusive in favor of exercise” and suggest the necessity for further research. The authors recognized mentioned uncertainty by applying the GRADE criteria, but the final inference missed this relevant point.

In addition to the placebo-controlled trials, Miller and colleagues [1] also investigated the efficacy of exercise compared to no-treatment control groups and usual care control groups. In both analyses, the authors detected a statistically significant effect of considerable magnitude of 1.02 (95% CI 0.67, 1.36) when comparing exercise to no treatment as well as 0.65 (95% CI 0.41, 0.89) when comparing exercise to usual care. Therefore, 0.94 as the mean estimate for the exercise-placebo comparison is not a negative outlier, which would justify the expectation of a null result.

The significance of the problem becomes obvious when academic articles are used by the popular press to publish misleading statements, such as this article from Bee [7] in *The Times* in which it is stated “that the benefits of exercise on easing discomfort for sufferers of chronic muscle pain are no better than sham placebo treatments such as fake pills, creams and injections.” This is an extremely harmful statement to make in regard to exercise therapy and also the consequence of the scientifically not convincing inferences made about the efficacy of exercise in this paper [1].
